# The prognostic value of microRNA-126 and microvessel density in patients with stage II colon cancer: results from a population cohort

**DOI:** 10.1186/s12967-014-0254-6

**Published:** 2014-09-10

**Authors:** Torben Frøstrup Hansen, Sanne Kjær-Frifeldt, Søren Morgenthaler, Thorarinn Blondal, Jan Lindebjerg, Anders Jakobsen, Flemming Brandt Sørensen

**Affiliations:** Department of Oncology, Vejle Hospital, part of Lillebaelt Hospital, Kabbeltoft 25, 7100 Vejle, Denmark; Department of Clinical Pathology, Vejle Hospital, part of Lillebaelt Hospital, Vejle, Denmark; Diagnostic Product Development, Exiqon A/S, Vedbæk, Denmark; Institute of Regional Health Research, University of Southern Denmark, Odense, Denmark

**Keywords:** Angiogenesis, Colon cancer, Duke’s B, microRNA-126, Microvessel density, Prognostic markers

## Abstract

**Background:**

Angiogenesis plays a pivotal role in malignant tumour growth and the metastatic process. We analysed the prognostic value of two angiogenesis parameters, microRNA-126 (miRNA-126) and microvessel density (MVD), in a population based cohort of patients operated for stage II colon cancer.

**Methods:**

A total of 560 patients were included. Analyses were performed on formalin fixed paraffin embedded tissue from the primary tumours. The analysis of miRNA-126 expression was performed by qPCR. Microvessels were visualised by CD105 and quantified in hot spots using a light microscope. The analyses were correlated with recurrence-free cancer specific survival (RF-CSS) and overall survival (OS).

**Results:**

Low miRNA-126 expression was significantly correlated to T4, high malignancy grade, tumour perforation, fixation, and the presence of microsatellite instability. A prognostic impact on OS was detected in the simple analysis favouring patients with high miRNA-126 expression p = 0.03, and borderline significance as to RF-CSS, p = 0.08. The impact on OS demonstrated borderline significance in a following multiple Cox regression analysis, hazard ratio 0.76 (95% confidence interval, 0.58-1.00), p = 0.051. The MVD estimate was not associated with either RF-CSS, p = 0.49, or OS, p = 0.94.

**Conclusion:**

The current population based study of patients operated for stage II colon cancer demonstrated correlations between several prognostic unfavourable characteristics and miRNA-126 and argues for a possible prognostic impact on overall survival. An influence on survival by the MVD estimate was not detected.

## Background

Evidence seems to support administration of adjuvant chemotherapy to patients with stage III colon cancer [1,2], while the indication in stage II disease is questionable [3]. The clinical problem of stage II colon cancer is to identify the patients in high risk of recurrence (approx. 20%) in order to offer them adjuvant chemotherapy with curative intent [4]. Currently, the selection of these patients is based on a number of surgical and pathological characteristics: Tumour perforation, bowel obstruction, poorly differentiated histology, pT4 tumours, vascular invasion, perineural invasion, lymphatic invasion and inadequate lymph node assessment [5]. These characteristics have all been related to poor outcome, but a clinical benefit of adjuvant chemotherapy for patients harbouring them has not been documented [3]. This calls for the identification of additional prognostic biomarkers.

Angiogenesis is a classical hallmark of cancer [6] and a prerequisite for the continuous growth of the malignant tumour, and simultaneously it provides an escape route for invasive tumour cells to metastasize [7]. This is reflected in studies demonstrating a relationship between aspects of angiogenesis and the prognosis of patients with cancer. The microvessel density (MVD), which is based on the morphological visualisation and quantification of blood vessels, represents an aspect and a possible prognostic value in colorectal cancer (CRC) [8]. The importance of MVD has been known for two decades [9] and while the majority of the literature seems to support MVD as having prognostic value in various types of cancer, counting of microvessels has never been integrated in the clinic. The reasons may be difficulties in reproducing results, the subjective nature of the assessment, and the fact that the majority of the literature is based on small, or medium sized, retrospective studies. A prospectively planned study of a large population based cohort of patients with stage II colon cancer may provide a definitive and valid evaluation of the clinical potential of this biomarker.

MicroRNAs (miRNAs) are small non-coding RNAs with the ability to regulate gene transcripts at a post transcriptional level [10,11]. The role of miRNAs as regulators of cellular homeostasis is constantly growing. Some miRNAs, called angio-miRs, are involved in the angiogenic process [12,13], where especially miRNA-126 seems to play a pivotal role. The main part of the literature supports an endothelial cell (EC) specific expression of this miRNA [14,15], although expression in cancer cells also has been reported [16]. These characteristics point towards miRNA-126 as a potential biomarker of angiogenesis. Studies have argued for a prognostic importance of miRNA-126 in different solid tumours [16-20], including CRC [21-23], but whether miRNA-126 is positively or negatively associated with survival is less clear and its role in stage II colon cancer in particular is largely unknown [22]. The analysis of miRNAs in stage II colon cancer holds the potential of adding a molecular biomarker to the existing panel of surgical and pathological characteristics.

The aim of this prospectively planed study was to analyse the prognostic impact of MVD and miRNA-126 in a population based cohort of patients operated for stage II colon cancer.

## Methods

Reporting in this study is in accordance with the REMARK [24] and BRISQUE [25] criteria.

### Patient population

**Figure 1:**
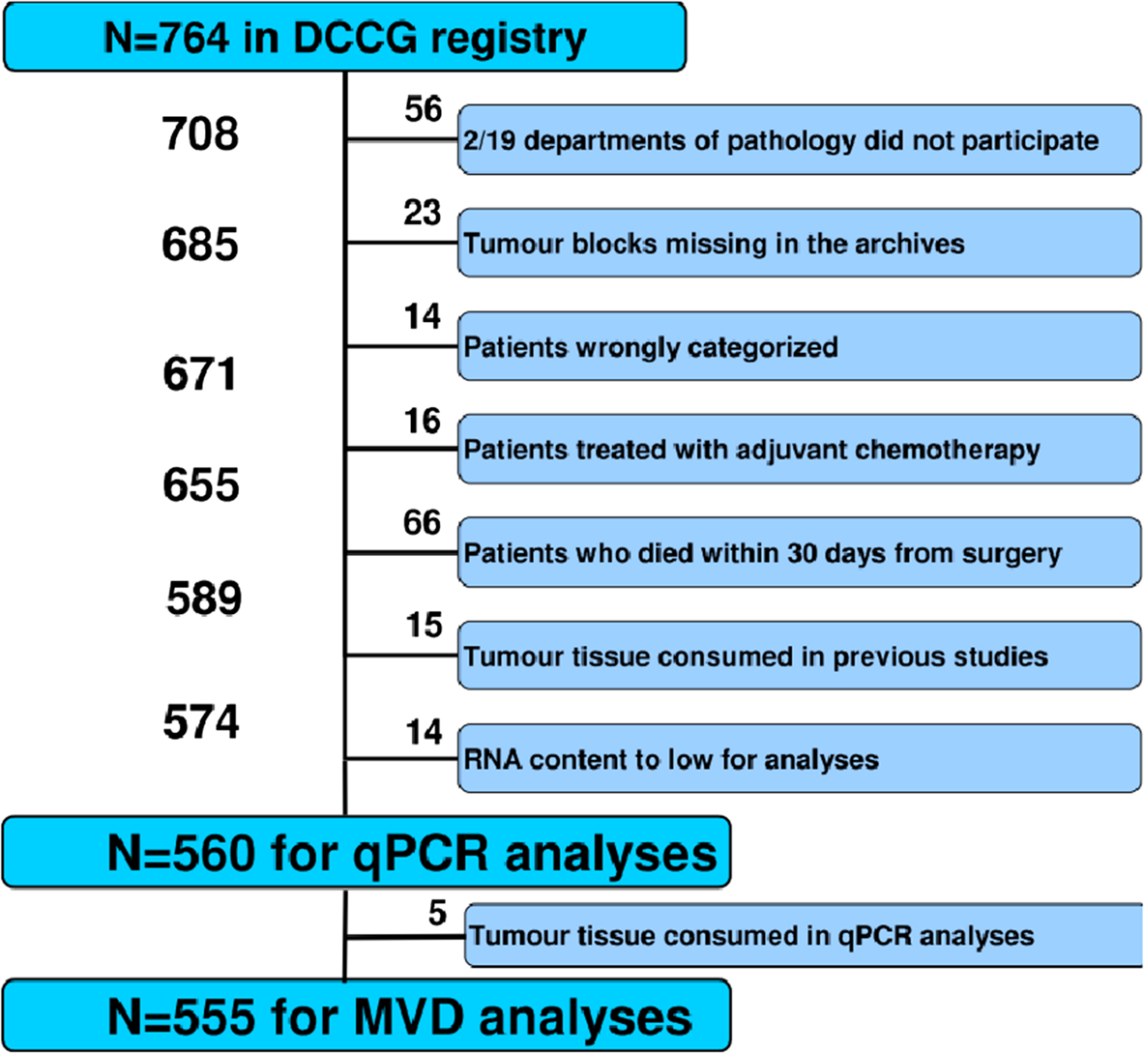
**Consort diagram of the study population.** DCCG: Danish Colorectal Cancer Group.

The patient population, follow-up, and sources of data have previously been described in detail [26]. In brief, a population cohort of patients surgically resected for stage II colon cancer was identified in the Danish Colorectal Cancer Group (DCCG) database, in which surgical and pathological data are collected prospectively. The entire cohort from 2003 constituted 764 patients. Participation in the study was high (~93%) leaving a representative cohort of the population. A flowchart of the study population is presented in Figure 1. A total of 560 patients was available for the present study. The study was approved by the Regional Scientific Ethical Committee for Southern Denmark (S-20090049) and The Danish Data Protection Agency according to Danish law.

### Samples

Histological samples from the resected colon tumours followed routine formaldehyde fixation and paraffin embedding (FFPE) and were transported and stored at room temperature. The median storage duration from archiving to analysis was 8.5 years. One FFPE tissue block containing the deepest invasive front was used from each patient to assess the pathological characteristics as previously described [26]. Four and six μm thick tissue sections were used for MVD and miRNA-126 analyses, respectively. Tissue sections were cut from the same FFPE tissue block but not adjacently, as several studies were carried out in parallel on the same study population.

### miRNA-126 analyses

MicroRNA quantification was carried out by Exiqon A/S (Vedbaek, Denmark). RNA was extracted in accordance with an optimized Qiagen FFPE purification protocol. Afterwards, 10 ng RNA was reverse transcribed (RT) in 10 μl reactions using the miRCURY LNA™ Universal RT miRNA PCR, Polyadenylation and cDNA synthesis kit (product no. 203301, Universal cDNA Synthesis Kit, Exiqon). cDNA was diluted X100 and assayed in 10 μl PCR reactions according to the miRCURY LNA™ protocol. MicroRNA-126 (product no. 204227 (hsa-miR-126-3p)), miRNA-16 (product no. 205702 (hsa-miR-16-5p)), miRNA-103 (product no. 204063 (hsa-miR-103a-3p)), and negative controls were assayed by qPCR on the microRNA Ready-to-Use PCR, Pick-n-Mix panel. Amplification was performed in a LightCycler® 480 Real-Time PCR System (Roche) in 384 well plates. Amplification curves were analyzed using the Roche LC software, both for determination of crossing point (Cp) and for melting curve analysis.

A pre-specified quality control removed reactions with several melting points, reactions with melting points not within assay specification, with amplification efficacy below 1.6, and with Cp within 5 Cp of the negative control reactions. Furthermore, an RNA spike-in control (Sp6) was added in the RT reaction in order to evaluate both the RT reaction and the following qPCR reaction. All except 14 samples (see Figure 1) passed quality control and the results showed overall similar Cp values indicating successful RT-qPCR analyses. Analyses were performed over three working periods. The results demonstrated comparable sample quality (samples were very similar in miRNA content), meaning that they had been processed reproducibly. The initial batch was analysed in duplicate. The variation in the system was very low with coefficient of variation percentages of 2.0, 1.2, and 1.7 for miRNA-16, miRNA-103, and miRNA-126, respectively, and the subsequent batches were consequently analysed once only.

The normalisation procedure was optimized for the present analysis to correct for potential differences in samples. The average values of miRNA-16 and miRNA-103 were used as normalisation factors. An alternative normalisation procedure was also considered using “global” miRNA content, which includes an additional 8 miRNAs with a presumable relationship to CRC carcinogenesis. This strategy, however, resulted in a higher degree of diversity between the replicates and was consequently abandoned. The miRNA-126 estimates are relative values normalised to the expression of other miRNAs as stated above and are thus presented without a dimension.

### CD105 and caldesmon immunostaining

Staining was performed by antibodies against CD105 and caldesmon in order to differentiate between immature and more mature microvessels. Tissue sections, 4 μm thick, were initially mounted on coated slides and dried for half an hour at 60°C and then overnight at 37°C. Deparaffinisation was performed in estisol for 10 min. at room temperature and followed by rehydration in graded alcohol solutions (99-70%). Blocking of endogenous peroxidase was achieved by adding hydrogen peroxide 3% for 5 min. Using manual procedures, antigens were unmasked by microwave oven heat-induced epitope retrieval, using a TEG buffer (TRIS 10 mM, EGTA 0.5 mM, Titriplex®-VI, Darmstadt, Germany) at pH 9 for 10 min at 1000 W and 15 min at 440 W. Tris-buffered saline (TBS)/Tween pH 7.6 was added for 5 min after cooling at room temperature. The tissue sections were then incubated overnight at 4°C with the anti-CD105 antibody (rabbit polyclonal, Thermo Scientific/AH Diagnostics) used in a 1:200 dilution.

Incubation with the anti-caldesmon antibody for 40 min (mouse monoclonal, IgG1, Dako, Clone h-CD, 1:50 dilution) and visualisation was performed on a Dako Autostainer Link 48. Visualisation was achieved using a Polymer cocktail: PowerVision Poly-AP anti-mouse IgG (AH Diagnostics) for Caldesmon, and EnVision + System-HRP(DAB), anti-rabbit (Dako) for CD105, for 40 min. This was followed by DAB + (Dako) for 12 min and Permanent Red Kromogen Kit (Cellmarque/AH Diagnostics) for 15 min. Nuclear staining with Mayers haematoxylin solution was performed on a Tissue-Tek Prisma machine and the staining intensity was enhanced by the addition of copper sulphate (0.5%).

Visualisation of CD105 and caldesmon was not possible in 5 samples due to insufficient amount of tumour tissue (see Figure 1) following the miRNA-126 analysis. Test for specificity was carried out by processing histological slides in parallel, in which the primary antibodies were omitted.

### Microvessel counting

**Figure 2:**
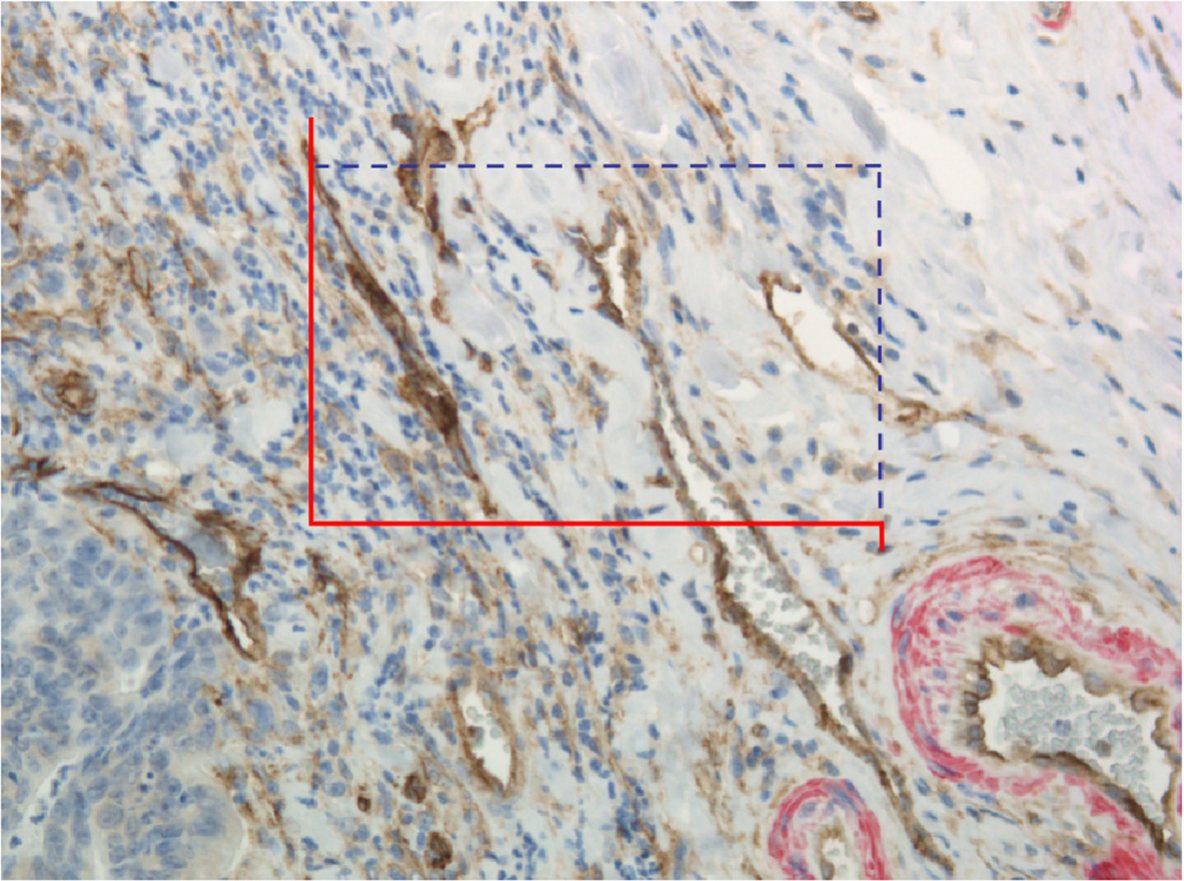
**Immunohistochemical expression.** CD105 positive structures (brown) with endothelial cell morphology. Surrounding positive caldesmon staining (red) is seen in more mature blood vessels. The unbiased counting frame is illustrated. Countable structures inside the frame or in contact with the non-solid blue line, and without contact with the red exclusive line, were counted and in this case 7 structures were counted. (Original magnification x 200).

The microvessel counting procedure and the interobserver variability of the analysis have previously been described in detail [27]. In brief, tumour sections were subjectively investigated for hot-spots at 40X and 100X, and counting was performed in three hot-spots at 200X in the microscope oculars using an inserted unbiased counting grid [28] with an area of 0.07 mm^2^. Random areas rich in vascularisation were chosen by the investigator in case 3 hot spots could not be identified. A representative example is provided in Figure 2. The mean MVD was used for the later analysis. Any brown CD105 positive (without any caldesmon positive smooth muscle cells around it) stained EC or structures with endothelial morphology clearly separated from adjacent microvessels by tumour cells and/or stromal elements were considered a single countable immature microvessel. The detection of vessel lumen was not a requirement. Hot-spots near tumour necrosis, ulcerations, and section edges were avoided.

Microvessels were counted by one observer (TFH) at the invasive tumour front without knowledge of patient outcome.

### Statistics

The Wilcoxon rank sum test was used for comparison of medians. Linear regression analysis was used to investigate the linear association between continuous variables. The prognostic value of variables was analysed by the log rank test, and survival curves were illustrated by the Kaplan-Meier method. Simple Cox regression analysis was used to estimate the hazard ratio of individual potential prognostic variables, and variables with p-values below 0.1 were included in a multiple Cox regression analysis. Overall survival (OS) was defined as time from operation until death of any course. Patients with stage II colon cancer have a rather high survival probability, which underlines the importance of cancer specific survival end-points in studies addressing disease related risk factors. Accordingly, recurrence free cancer specific survival (RF-CSS), defined as time from operation until documented tumour recurrence or death from colon cancer, constituted the second endpoint. Other malignancies and death from other courses than colon cancer were censored from this analysis.

All statistical calculations were carried out using the NCSS statistical software (NCSS Statistical Software, Kaysville, UT 84037, USA, version 2007). P values < 0.05 were considered significant, and all tests were two-sided.

## Results

Follow-up ended December 31st 2010. Of the 560 patients, 119 experienced recurrence and 256 died (111 of colon cancer and 145 from other reasons than cancer) in the 7-year follow-up period.

### Patient characteristics

Both miRNA-126 and MVD were analysed in all samples except five in which MVD analysis was not possible as previously explained. Patient characteristics, miRNA-126 and MVD data are shown in Table 1. The median miRNA-126 expression was significantly lower in T4 tumours, and in tumours with high malignancy grade, perforation and fixation, or microsatellite instability (MSI). Patients with low miRNA-126 expression (below the median) were characterised by having T4 tumours (18%), high malignancy grade (23%), perforation (12%), fixation (22%), and MSI (34%), respectively. A significantly lower median MVD was detected in tumours with a high malignancy grade as well.

**Table 1:** **Patient characteristics according to miRNA-126 and MVD**

**Parameter**	**Number**	**miRNA-126**	**p-value**	**MVD**	**p-value**
	**(n = 560) (%)**	**(n = 560)**		**(n = 555)**	
		**Median (95% CI)**		**Median (95% CI)**	
**Sex**					
Male	241 (43)	2.49 (2.38-2.59)	0.61	10.7 (10.3-11.0)	0.17
Female	319 (57)	2.47 (2.40-2.53)		10.3 (10.0-10.7)	
**Age**, median 74					
≥74	280 (50)	2.49 (2.42-2.56)	0.31	10.7 (10.3-11.0)	0.11
<74	280 (50)	2.44 (2.34-2.53)		10.3 (10.0-10.7)	
**T category**					
T4	73 (13)	2.20 (2.03-2.42)	**0.0004**	10.3 (10.0-11.3)	0.59
T3	487 (87)	2.50 (2.44-2.56)		10.3 (10.0-10.7)	
**Malignancy grade**					
High*	107 (19)	2.33 (2.17-2.48)	**0.01**	10.0 (9.3-10.7)	**0.03**
Medium + Low	453 (81)	2.49 (2.43-2.56)		10.7 (10.3-10.7)	
**Localisation**					
Right	286 (51)	2.48 (2.39-2.54)	0.45	10.3 (10.0-10.7)	0.75
Left	274 (49)	2.48 (2.38-2.56)		10.3 (10.0-10.7)	
**Perforation**					
Yes	49 (9)	2.36 (2.05-2.42)	**0.003**	11.0 (10.0-12.0)	0.19
No	511 (91)	2.49 (2.43-2.56)		10.3 (10.0-10.7)	
**Fixation**					
Yes	100 (18)	2.36 (2.12-2.47)	**0.0009**	10.7 (10.3-11.3)	0.15
No	460 (82)	2.50 (2.43-2.57)		10.3 (10.0-10.7)	
**Lymph nodes** ^a^					
≥12	251 (45)	2.49 (2.39-2.54)	0.96	10.7 (10.0-11.0)	0.10
<12	306 (55)	2.47 (2.39-2.56)		10.3 (10.0-10.7)	
**Neuronal invasion**					
Yes	47 (8)	2.38 (2.16-2.59)	0.66	10.0 (9.0-10.7)	0.29
No	513 (92)	2.48 (2.42-2.53)		10.3 (10.3-10.7)	
**Vascular invasion**					
Yes	67 (12)	2.48 (2.31-2.65)	0.81	10.3 (10.0-11.0)	0.98
No	493 (88)	2.48 (2.42-2.53)		10.3 (10.0-10.7)	
**MSI status**					
MSI	159 (28)	2.30 (2.17-2.46)	**0.003**	10.3 (10.0-11.0)	0.83
MSS	401 (72)	2.53 (2.46-2.59)		10.3 (10.0-10.7)	

CI: confidence interval; miRNA-126: microRNA-126; MSI: microsatelite instability; MSS: microsatelite stable; MVD: microvessel density.

^a^Not assessed for all patients.

*Including mucinous and sigillocellular adenocarcinomas.

Significant p-values are highlighted in bold.

### Correlation between the investigated parameters

**Figure 3:**
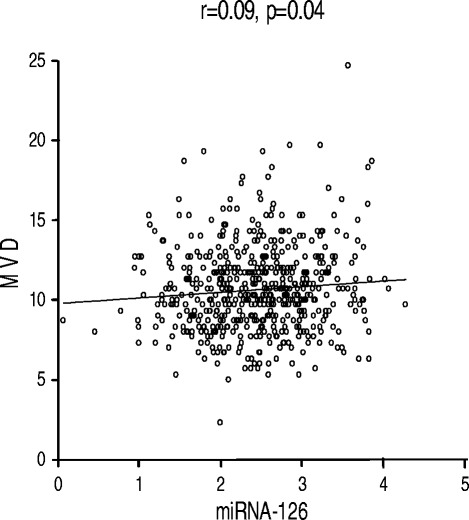
**Linear regression analysis.** MicroRNA-126 (miRNA-126) and microvessel density (MVD).

The correlation between miRNA-126 expression and MVD is shown in Figure 3. A weak, although significant, positive correlation was demonstrated, r = 0.09, p = 0.04.

### Simple survival analyses

**Figure 4:**
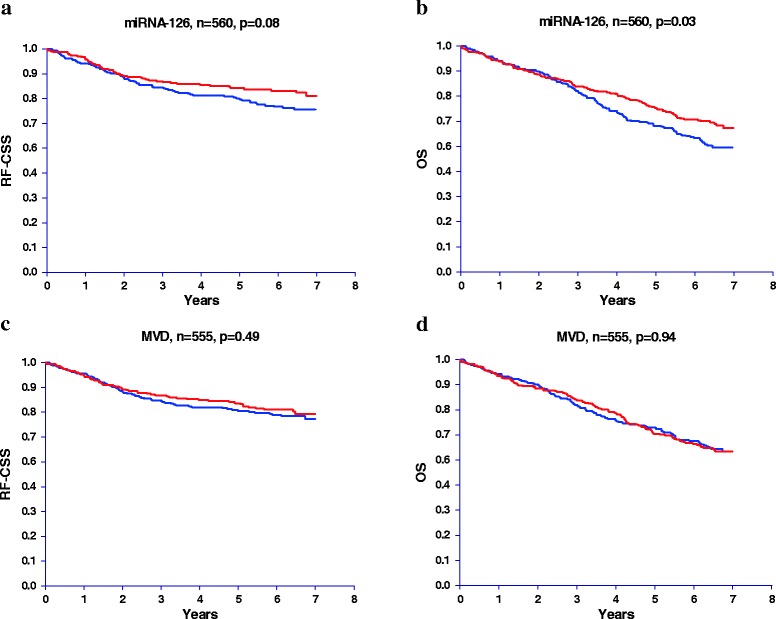
**Recurrence free cancer specific survival (RF-CSS) and overall survival (OS) according to microRNA-126 (miRNA-126) expression and microvessel density (MVD). (a)** miRNA-126 (RF-CSS), **(b)** miRNA-126 (OS), **(c)** MVD (RF-CSS), and **(d)** MVD (OS). Red lines represent patients with miRNA-126 expression or MVD above the median.

The relationships between miRNA-126, MVD, RF-CSS, and OS are shown in Figure 4 (a-d). Patients were grouped according to the median miRNA-126 and MVD, respectively. A significant relationship between miRNA-126 and OS was demonstrated (p = 0.03) favouring patients with high miRNA-126 expression. A similar separation of the RF-CSS curves was detected according to miRNA-126 expression, although this difference only reached borderline significance (p = 0.08). No differences were observed in RF-CSS and OS according to MVD.

The corresponding simple Cox Regression analyses for miRNA-126, MVD, and patient and tumour characteristics are shown in Tables 2 and 3. Significant relationships with RF-CSS were demonstrated for patients with T4 tumours, tumour perforation and/or fixation, or neuronal and/or vascular invasion. Gender, age, T4, perforation, fixation, vascular invasion, and miRNA-126 were significantly related to OS.

**Table 2:** **Cox regression analysis, recurrence free cancer specific survival (n = 560 in the multiple analysis)**

	**Simple analysis**			**Multiple analysis**		
	**HR**	**95% CI**	**p-value**	**HR**	**95% CI**	**p-value**
**Sex**						
Female	1					
Male	1.16	0.81-1.67	0.41			
**Age**, median 74						
<74	1					
≥74	1.14	0.80-1.64	0.47			
**T category**						
T3	1			1		
T4	3.65	2.46-5.43	**<0.0001**	2.69	1.74-4.16	**<0.0001**
**Malignancy grade**						
Medium + Low	1			1		
High*	1.49	0.98-2.27	0.06	1.42	0.92-2.18	0.11
**Localisation**						
Right	1					
Left	1.12	0.78-1.60	0.54			
**Perforation**						
No	1			1		
Yes	3.66	2.34-5.73	**<0.0001**	2.57	1.54-4.30	**0.0003**
**Fixation**						
No	1			1		
Yes	2.49	1.69-3.67	**<0.0001**	1.32	0.83-2.10	0.23
**Lymph nodes**						
≥12	1					
<12	0.85	0.59-1.22	0.37			
**Neuronal invasion**						
No	1			1		
Yes	2.05	1.24-3.38	**0.005**	1.46	0.84-2.55	0.18
**Vascular invasion**						
No	1			1		
Yes	2.00	1.27-3.16	**0.003**	1.61	1.00-2.61	0.05
**MSI status**						
MSI	1					
MSS	1.39	0.90-2.15	0.14			
**miRNA-126**						
<median	1			1		
>median	0.72	0.50-1.04	0.08	0.96	0.66-1.40	0.83
**MVD**						
<median	1					
>median	0.88	0.61-1.26	0.49			

HR: hazard ratio; CI: confidence interval; MSI: microsatellite instability; MSS: microsatelite stable; miRNA-126: microRNA-126; MVD: microvessel density.

*Including mucinous and sigillocellular adenocarcinomas.

Significant p-values are highlighted in bold.

**Table 3:** **Cox regression analysis, overall survival (n = 560 in the multiple analysis)**

	**Simple analysis**			**Multiple analysis**		
	**HR**	**95% CI**	**p-value**	**HR**	**95% CI**	**p-value**
**Sex**						
Female	1			1		
Male	1.41	1.08-1.84	**0.01**	1.68	1.28-2.19	**0.0002**
**Age**, median 74						
<74	1			1		
≥74	2.39	1.80-3.16	**<0.0001**	2.58	1.94-3.42	**<0.0001**
**T category**						
T3	1			1		
T4	1.99	1.43-2.78	**0.0001**	1.76	1.24-2.49	**0.002**
**Malignancy grade**						
Medium + Low	1					
High*	1.22	0.88-1.69	0.24			
**Localisation**						
Right	1					
Left	0.96	0.73-1.24	0.74			
**Perforation**						
No	1			1		
Yes	1.80	1.20-2.72	**0.005**	1.49	0.96-2.32	0.08
**Fixation**						
No	1			1		
Yes	1.60	1.17-2.18	**0.004**	1.26	0.89-1.78	0.20
**Lymph nodes**						
≥12	1					
<12	1.06	0.82-1.39	0.64			
**Neuronal invasion**						
No	1					
Yes	1.19	0.76-1.87	0.44			
**Vascular invasion**						
No	1			1		
Yes	1.60	1.12-2.30	**0.01**	1.47	1.02-2.11	**0.04**
**MSI status**						
MSI	1					
MSS	0.92	0.69-1.24	0.59			
**miRNA-126**						
<median	1			1		
>median	0.75	0.57-0.98	**0.03**	0.76	0.58-1.00	0.051
**MVD**						
<median	1					
>median	1.01	0.77-1.32	0.94			

HR: hazard ratio; CI: confidence interval; MSI: microsatellite instability; MSS: microsatelite stable; miRNA-126: microRNA-126; MVD: microvessel density.

*Including mucinous and sigillocellular adenocarcinomas.

Significant p-values are highlighted in bold.

### Multiple Cox regression analyses

A cut-off significance level of 0.1 was pre-specified for a parameter to be included in the multiple Cox regression analysis. Category T4 and tumour perforation remained significant as to RF-CSS, and gender, age, T4, and vascular invasion demonstrated significant influence on OS. An independent influence by miRNA-126 expression on RF-CSS could not be demonstrated (p = 0.83), while borderline significance was achieved in the OS analysis, HR 0.76 (95% CI, 0.58-1.00), p = 0.051 (Tables 2 and 3).

## Discussion

The structural and functional abnormalities of tumour associated blood vessels might select for tumour cell clones with a high metastatic potential [29]. Thus, assessing angiogenesis in stage II colon cancer may identify patients with risk of disease recurrence, and aid selecting candidates for adjuvant therapy/close observation. In this study, performed on a population based cohort of patients with stage II colon cancer, miRNA-126 expression was documented to correlate with several characteristics defining high-risk patients and a possible influence on OS is suspected. The MVD estimate was not correlated with prognosis.

The results demonstrated close correlations between miRNA-126 expression and other prognostic markers. The study by Li *et al.* demonstrated a similar significant relationship between high malignancy grade and low miRNA-126 expression [30], while two other studies did not identify any significant relationships [21,31]. This may be caused by rather small sample sizes (66 < N < 110) and most importantly heterogeneous samples including stage I through IV disease. Nevertheless, the present relationships all point towards an association between low miRNA-126 expression and variables defining increased tumour growth as demonstrated in several different solid tumours, including CRC [30,32].

A rather weak, although significant positive correlation between miRNA-126 expression and MVD was detected in the present study. A similar positive relationship between these two parameters has been seen in previous studies of patients with CRC [31,33], while negative correlations have been detected in other solid tumours [19,20]. Possible reasons for these divergent results are; inclusion of multiple different tumour types, disease stages, comparability of samples (non-adjacent sections as in this study), analytic methodology, ethnicity, and technical differences between quantification of immunohistochemical estimates and the PCR outcome.

In the simple RF-CSS analysis borderline significance was detected favouring patients with high miRNA-126 expression. As expected due to the correlation between miRNA-126 expression and several of the included parameters we did not see this difference in the multiple Cox regression analyses. Thus, miRNA-126 seems to be related to many of the prognostic unfavourable characteristics driving tumour growth, but it did not contribute independently to RF-CSS. The current literature on CRC regarding miRNA-126 and prognosis reports divergent results. Two studies on small subgroups of patients (N = 28 and 37) with stage II microsatellite stable colon cancer demonstrated a relationship between low miRNA-126 and favourable prognosis [22,31], one study based on 110 patients with stage I through IV CRC did not show any relationship [21], and finally two studies on the same cohort of 89 patients with stage IV disease presented a relationship between high miRNA-126 expression and a favourable prognosis [23,33]. Despite these divergent results, current evidence, taking the present results into account, suggests a favourable prognosis for patients with high miRNA-126 expressing tumours, which is in line with the concept of miRNA-126 functioning as a tumour suppressor [34].

MicroRNA-126 expression demonstrated a significant difference in favour of improved OS in patients with high expressing tumours in the simple analysis and this difference remained almost unchanged in the multiple Cox regression analysis. In an analysis dominated by non-cancer specific events, this relationship may indicate a prognostic importance of miRNA-126 to survival in general. Assuming that there is some degree of equilibrium between miRNA-126 levels in the tumour and the blood circulation, these results could be explained by the proposed impairment of neovascularisation following ischemic vascular events such as acute myocardial ischemia, which is one of the most common non-cancer causes of death described in relation to miRNA-126 levels [35,36].

Normalisation of qPCR data in the present study was based on the average of miRNA-16 and miRNA-103 as described previously, which is in line with previous reports [37,38]. RNUs were not considered for normalisation due to concerns about their stability in FFPE tissue samples and their possible relationship with clinicopathological characteristics such as prognosis [39].

The MVD estimate was not correlated to either RF-CSS or OS in the present population study. Over the last two decades around thousand of studies have been published investigating the role of microvessels in human malignancies; in CRC isolated close to a hundred can be compiled. Many of the studies have addressed the prognostic role of different microvessel estimates (MVD being the most common one) and the majority seems to support a prognostic value of these estimates [8,40,41]. However, conflicting results do exist and it is worth noting that the largest studies we identified included more than 200 patients (range, 204–235) and none were able to demonstrate a significant relationship between MVD and prognosis [42-44]. Numerous methodological reasons potentially biasing the results in MVD studies have previously been addressed [27], and the subjective nature of this estimate along with difficulties in reproducing the results may be the main reasons. A certain degree of publication bias may also be suspected after the many years in focus. Nevertheless, our current findings, from the first population based study analysing the prognostic value of MVD in more than 500 patients, does not indicate any prognostic value of this classical estimate. It is, however, important to stress that other histology based estimates of angiogenesis, applying different methodologies than the subjective scoring of MVD in hot-spots, may still provide clinical information of prognostic relevance.

## Conclusions

The current population based study included more than 90% of the patients operated for stage II colon cancer in Denmark in 2003 and is thus highly representative and considered unbiased for selection. The study holds two major conclusions:

1. The EC specific miRNA-126 demonstrated significant correlations to several prognostic unfavourable characteristics driving tumour growth without contributing independently to cancer specific survival. However, a possible influence on survival in general is speculative. These results strengthen the relevance of addressing angiogenesis when grading colon tumours as high or low risk. The ideal way to quantify angiogenesis is, however, still debatable. The relationship with overall survival suggests a possible therapeutic and biomarker potential deserving further attention.
2. The classical marker of angiogenesis, MVD, demonstrated no relationship with survival and the clinical potential of this marker in selecting patients with high risk stage II colon cancer seems limited. This observation calls for alternative ways of quantifying tumour associated angiogenesis.
